# Histological characteristics of bone in-growth of proximal humeral implants with different spatial structures

**DOI:** 10.18632/aging.202391

**Published:** 2021-01-20

**Authors:** Zhe Xue, Zheng Pei, Hui Zhang, Chong Tang, Junxiu Jia, Kun Zhang, Keshi Zhang, Luning Wang, Zhenpeng Guan

**Affiliations:** 1 Department of Orthopedics, Peking University Shougang Hospital, ShiJingshan 100144, Beijing, P.R. China; 2 Beijing Advanced Innovation Center for Materials Genome Engineering, School of Materials Science and Engineering, University of Science and Technology Beijing, Haidian 100083, Beijing, P.R. China

**Keywords:** titanium alloy implant, trabecular metal, proximal humerus, bone in-growth

## Abstract

This study compares the longitudinal histological characteristics of proximal humeral implants with different spatial structures in rabbits. Thirty skeletally-mature male rabbits were divided into a trabecular structure group and regular hexahedron structure group according to the different spatial structures of a biological titanium alloy screw inserted into the greater tuberosity of the proximal humerus. Samples were collected 3, 6, and 12 weeks after the implantation surgery. Histological results showed that the amount of bone in-growth in the porous cavity of the screw implant increased over time. Quantitative analysis showed there was significantly more bone in-growth in the trabecular structure group than the classic structure group 3 weeks (25.4% ± 6.9% vs 19.6% ± 3.7%, *P* < 0.05) and 6 weeks (31.2% ± 1.7% vs 26.9% ± 5.3, *P* < 0.05) after the implantation surgery. No significant difference was detected between the two groups 12 weeks after the surgery (41.7% ± 2.5% vs 39% ± 4.1%, *P* > 0.05). Our data found that bone in-growth significantly differed among the three time points (*P* < 0.05) in both groups, but not between the implants with different spatial structures 12 weeks after the surgery.

## INTRODUCTION

Shoulder arthroplasty is mainly used for treating elderly patients with complex proximal humeral fractures, rotator cuff tears, severe osteoarthritis, rheumatoid arthritis, severe traumatic arthritis, and other severe shoulder injuries. There are different clinical reports on the postoperative outcomes of shoulder arthroplasty. The most common postoperative complication is a malunion of the greater tuberosity of the humerus, which affects the postoperative clinical functions of the shoulder joint [1]. Currently, a porous-coated design is the commonly used artificial shoulder prosthesis. Although it improves the bone-prosthesis healing condition to a certain extent, there are still some defects, including the interfacial sheer force between the coating and the prosthesis, a low porosity and galvanic effect, which can affect the bone in-growth and the healing of greater tuberosity of proximal humerus [1, 2]. Previous studies have confirmed that the classic hexahedral porous titanium alloy implants, forged by electron beam melting technology, have the ability to overcome the shortcomings of a prosthesis with a traditional coating and accelerate the bone in-growth. Nevertheless, the hexahedral structure is different from the physiological structure of the human bone (trabecular structure) [1]. However, there are no studies that state whether implants with a trabecular structure, which is closer to the physiological structure of human bone, are more conducive to accelerating the speed and proportion of bone in-growth and obtaining better postoperative biomechanical properties.

In this study, New Zealand rabbits were used to as an animal model. Trabecular porous titanium alloy implants (study group) and classic porous titanium alloy implants (control group) were implanted into the rabbit’s greater tuberosity of proximal humerus. The purposes of this study were to: (1) determine whether the two titanium alloy implants with different porous structures have bone in-growth potential in the proximal humerus longitudinally, and (2) compare the histological characteristics of bone in-growth in the two different porous structure implants at different time points.

## RESULTS

### Animal model observation

All 30 rabbits were in good health after the operation and began to eat autonomously immediately the day of modeling. No infection was found near the wound during the observation period. The modeling operation showed that the shoulder joint’s local anatomical structure and relevant anatomical landmarks was similar to that of humans. This demonstrated that the establishment a the biological implant model in the greater tubercle of the shoulder joint is repeatable.

### General situation of bone in-growth areas in different spatial structures

**Figure 1:**
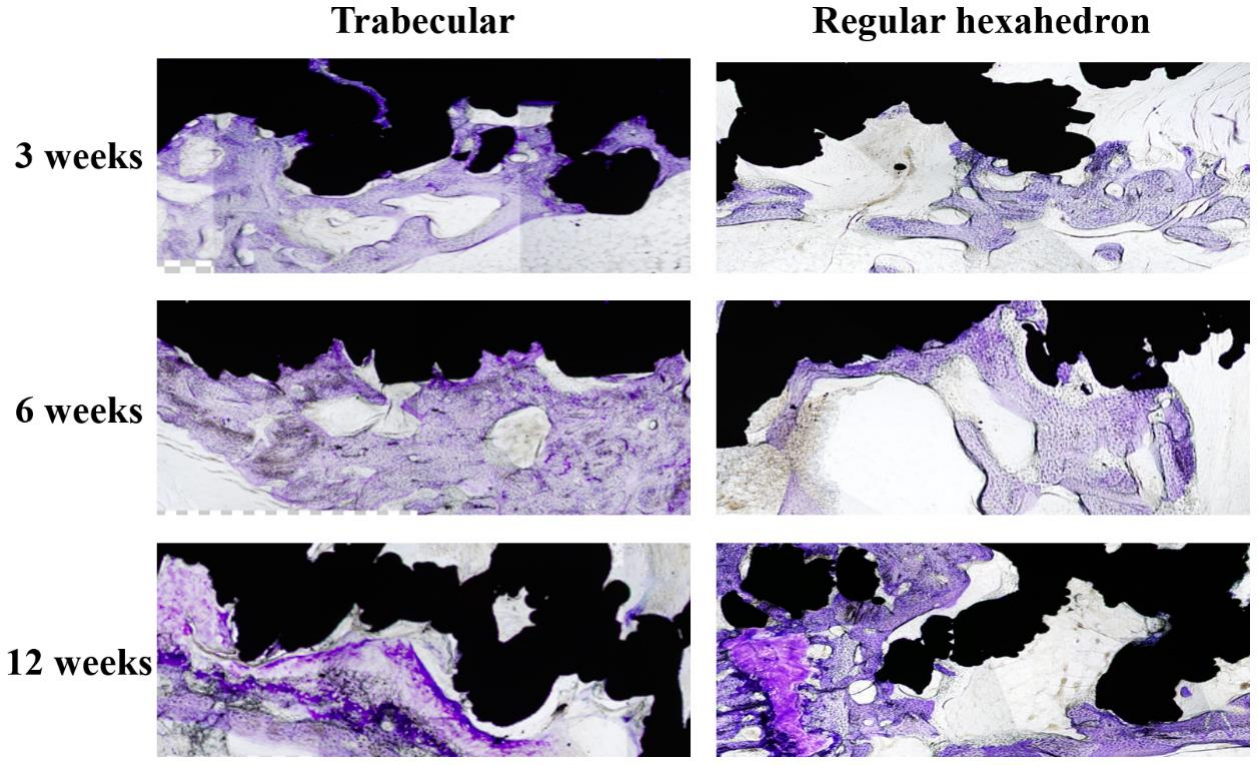
**The longitudinal comparison of the bone in-growth in different spatial structures screws (local feature).** The black area is the screw and the dyed area (purple) is the bone in-growth tissue. The distribution area and density of chondrocytes in the pore of the screw increased in both trabecular and regular hexahedron structure groups at the three follow-up time points (3, 6, 12 weeks).

A microscopic histological slice stain at 3, 6, and 12 weeks, see Figure 1, showed the amount of cartilage cells in both screws had positive growth trend and gradual expanding distribution.

### Longitudinal variation tendency of bone in-growth in different pore structure implants

The histological measurement results are shown in Table 1 and Table 2. These results show that the bone in-growth percentage area in the trabecular structure implant group was significantly larger than the classic structure control group at 3 and 6 weeks (*P* < 0.05), but no significant differences were found at 12 weeks (*P* > 0.05). Furthermore, there were significant differences within both of the two groups when intergroup pairwise comparisons were performed in both groups (*P* < 0.05) (Table 3).

**Table 1:** Bone in-growth results of regular hexahedron structure screw.

**Sample number**	**Total area of rectangle**	**Bone in-growth area**	**Percentage of bone in-growth area (S% =S_1_/S_0_ x 100%)**
	**(S_0,_ mm^2^)**	**(S_1_, mm^2^)**	
1	35.07	8.24	23.5
2	39.68	7.02	17.7
3	38.97	7.33	18.8
4	33.21	7.11	21.4
5	33.00	7.79	23.6
6	44.64	14.02	31.4
7	62.1	16.08	25.9
8	67.85	18.12	26.7
9	56.57	16.35	28.9
10	62.83	18.97	30.2
11	38.51	11.31	29.4
12	38.14	19.68	51.6
13	43.04	14.33	33.3
14	44.28	16.34	36.9
15	28.35	13.83	48.8

**Table 2:** Bone in-growth results of trabecular structure screw.

**Sample number**	**Total area of rectangle**	**Bone in-growth area**	**Percentage of bone in-growth area (S% =S_1_/S_0_ x 100%)**
	**(S_0_, mm^2^)**	**(S_1_, mm^2^)**	
1	34.01	10.11	0.30
2	40.23	10.53	0.26
3	39.07	11.25	0.29
4	35.31	10.01	0.28
5	34.80	10.76	0.31
6	42.53	15.13	0.36
7	55.16	19.69	0.35
8	62.65	21.53	0.34
9	54.87	19.69	0.36
10	59.88	21.79	0.34
11	40.53	17.32	0.43
12	39.22	18.68	0.48
13	41.04	17.31	0.42
14	43.68	18.14	0.42
15	44.66	17.89	0.40

**Table 3:** Results of bong in-growth areas of two different structure screws at three time points $(\bar{x}\pm s)$.

**Time points (weeks)**	**Percentage of bone in-growth area of regular hexahedron (%)**	**Percentage of bone in-growth area of trabecular (%)**	***P***
3	21.0±2.7	28.8±1.7	0.017*
6	28.6±2.3	35.6±3.7	0.000*
12	40.0±9.7	42.8±5.1	0.235

*P<0.05. Significant difference.

## DISCUSSION

Two findings stand out as being the most important. First, the bone in-growth percentage area in both implant groups presented an increasing trend in the greater tuberosity of proximal humerus longitudinally the rabbits. Second, although the bone in-growth speed of the trabecular structure implant was significantly higher than the implant with the classic structure within six weeks, the histological measurement confirmed the total area of bone in-growth in both groups’ spatial structure implants showed no significant difference at 12 weeks.

The porous coating design is the most commonly used artificial shoulder prosthesis in clinical practice. Although other coating materials could improve the bone-prosthesis healing to a certain extent, there are still problems that could prevent achieving the true purpose of bone in-growth, such as the sheer force of the material interface, a low porosity, and the galvanic effect [2]. Therefore, it was of great necessity to design and develop a prosthesis with a three-dimensional spatial structure to improve the bone-prosthesis healing condition and improve the biomechanical fixed strength. A new implant metal material, titanium alloy with a classic pore structure, made by electron beam melting technology using titanium alloy as raw material could replace previous implant materials. Previous studies confirmed that the screw with classic pore structure, formed by the regular arrangement and divergence of regular hexahedron mesh into surrounding space, had considerable bone in-growth characteristics [1]. Our study showed that the trabecular spatial structure implant, which was similar to the structure of cancellous bone, had the analogical bone in-growth potency of the hexahedral structure implant, with faster bone in-growth speed and a larger area early post-operation (within six weeks). This finding also provided a theoretical basis for the selection of endophytes with different pore structures in clinical treatments.

The main factors that currently affect the healing of the greater tuberosity of proximal humerus after total shoulder replacement included the age, gender, bone status, prosthesis position, rotator cuff quality, and prosthesis type of patient [3–7]. The main principle of the coating design was to promote the healing between the proximal part of the implant and the greater tuberosity by making multiple porous parts on the prosthesis, and theoretically improve the healing condition of the greater tuberosity after operation. However, a multicenter clinical study found that the healing rate of the greater tuberosity was still unsatisfactory [1, 7–12]. This study preliminarily confirms the potential and characteristics of different spatial structure materials, and provides the necessary theoretical and practical basis for improving the design of the proximal prosthesis and the healing rate of the greater tuberosity after operation for patients.

The two kinds of space structure materials in this study were stereoscopic divergent structures. We have preliminarily confirmed the advantages of the two space structure materials in terms of bone in-growth through the histological research. Compared with the hexahedral structure implant, the trabecular structure implant played a more prominent role in early postoperative bone in-growth. The corresponding clinical significance lies in patients’ early rehabilitation exercises after shoulder joint replacement. In this study, the rate of bone in-growth in the trabecular structure was significantly higher than that in the classic hexahedral structure within six weeks, but there was no significant difference at 12 weeks between the two different structures. Active auxiliary exercise is recommended to be performed 4-6 weeks after artificial shoulder replacement surgery for rehabilitation [13–15]. However, some literature has questioned if early rehabilitation exercise interferes with the healing process of the greater tuberosity of proximal humerus and if it can lead to postoperative failure [16]. According to the results of this study, if the trabecular structure implant is used during the operation, there could be better bone in-growth characteristics than the classic structure implant at the all-important early stage post-operation. Using a titanium alloy implant with a new trabecular structure may be a better choice for the surgeons in the future in order to enhance the biomechanical properties of the host’s implant and ensure early rehabilitation exercises do not affect the long-term outcome of the operation, while reducing the postoperative complications.

This research had some limitations. First, the sample size of the experimental animals was small, the feeding time was relatively short, and there were no long-term follow-up results. Second, there was a lack of three-dimensional quantitative research, such as the bone in-growth volume analysis, despite a single cross-section analysis (total area). Third, this study only conducted out a toluidine blue staining procedure. No other fluorescent protein staining study or biomechanical experiment confirmed the biomechanical properties of host-implant. Therefore, additional immune histochemical and biomechanical investigations are needed to confirm the histochemical and biomechanical properties of titanium alloy implants with different spatial structures.

This histological study confirmed that the bone in-growth area of different spatial structure implants in the greater tuberosity of proximal humerus in rabbit models increased longitudinally. The bone in-growth speed of the trabecular structure implant was significantly higher than that of the classic structure implant during the early stage post-operation (within six weeks). Nevertheless, the total area of bone in-growth in the two different spatial structures showed no significant difference at twelve weeks.

## MATERIALS AND METHODS

### Establishment of animal models

From August 2018 to May 2019, thirty skeletally-matured (four months old) male, New Zealand, big-ear white rabbits were randomly divided equally into two groups, a study group and control group, weighing between 2.53 kg and 2.71 kg. Each of the rabbits were fed in a single cage with common rabbit food and observed for two weeks before the surgery. They were confirmed to be healthy and without other disease. The disposal of animals conformed to the standards of medical ethics.

### Modeling

**Figure 2:**
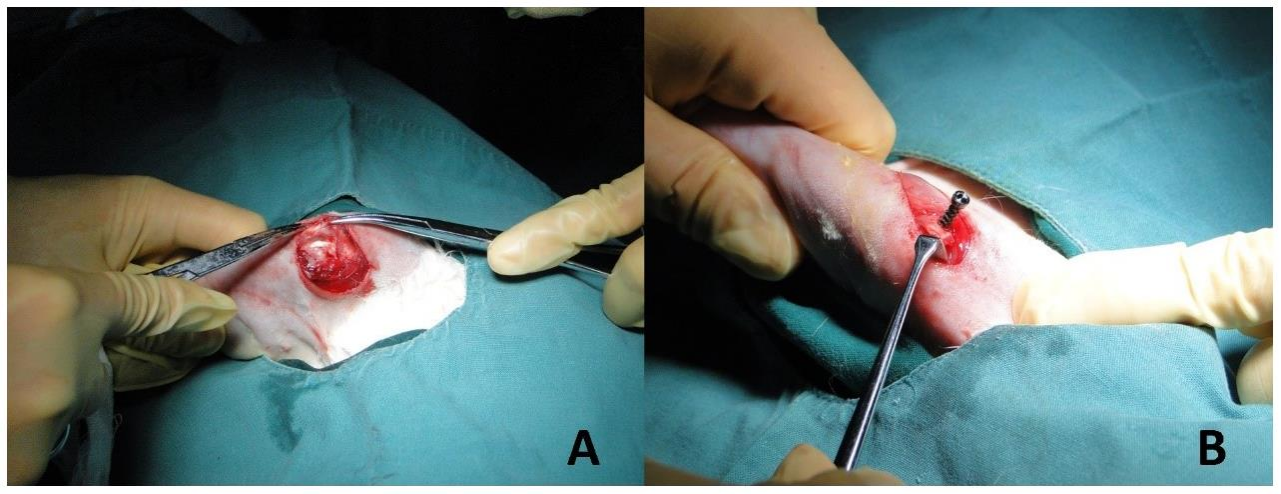
**Establishment of implant model.** (**A**) The deltoid muscle of the rabbit was bluntly dissected to reveal the greater tubercle of humerus. (**B**) Titanium alloy screws with different pore structures (trabecular pore structure in the study group and classical pore structure in the control group) were drilled in the long axis of the distal humerus approximately 120 degrees. The implant models of titanium alloy with different spatial structures of proximal humerus were established.

**Figure 3:**
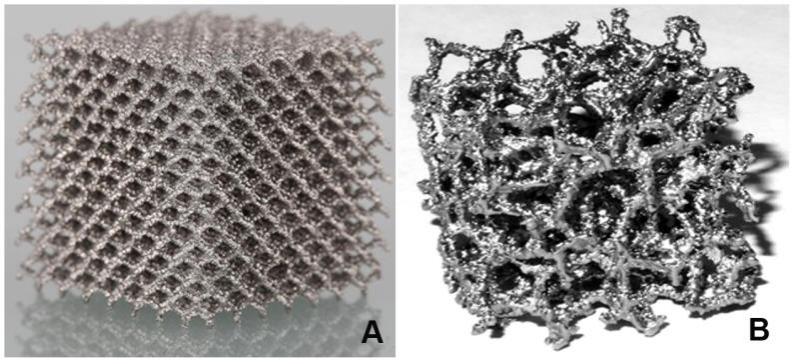
**Classical and trabecular pore structures.** (**A**) The plant structure in titanium alloy with classical pore structure was arranged in the surrounding space with regular hexahedral mesh as the base. (**B**) The plant structure in titanium alloy with trabecular pore structure was similar to normal human cancellous bone, which was called "cancellous bone like."

A Su-Mian-Xin II injection (speed of 0.2ml/kg) with intramuscular injection was used to provide general anesthesia on the experimental animals. The rabbits were placed in the left recumbent position after the successful administration of anesthesia. The rabbits were then prepped for operation by preparing the skin, performing routine disinfection, and draping the area. A 2 cm longitudinal skin incision was made at the shoulder joint. Blunt dissection of the deltoid muscle revealed the greater tubercle (Figure 2A). At the greater tuberosity, a 2.0 K-wire was drilled into the humerus passing through the center of the shaft, about 120 degrees from the long axis of the distal humerus. In the study group, titanium alloy screws with trabecular pore structure were inserted along the bone tunnel; the classical pore structure screws were inserted in the control group (Figure 3A, 3B). Additionally, we accomplished establishing titanium alloy implantation models with different spatial structures in the proximal humerus (Figure 2B). Lastly, the wound was stitched together layer-by-layer after examination to insure there was no active bleeding.

### Postoperative processing

**Figure 4:**
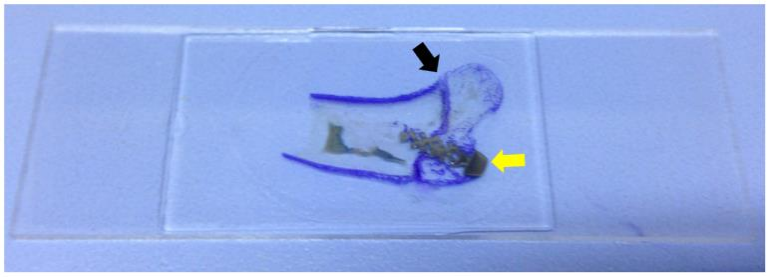
**Slice specimen of the bone in-growth.** The dyed area (purple, black arrow) is the greater tuberosity of proximal humerus. The grey area is the screw (yellow arrow).

**Figure 5:**
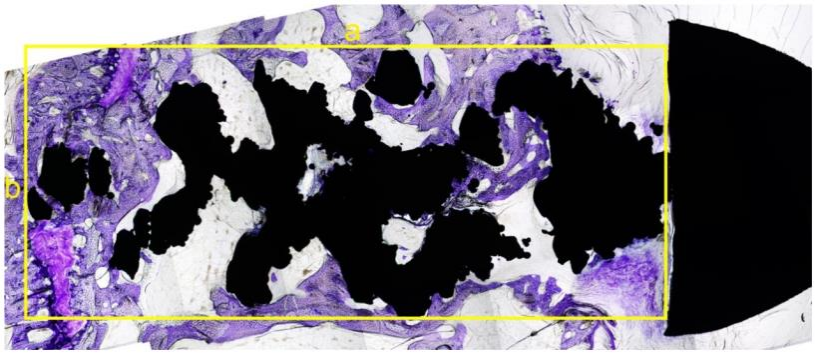
**The sagittal slice of histological section of the screw.** The black area was the screw (the nut is on the right) and the dyed area (purple) was the bone in-growth tissue. A rectangle was made that can contain part of the screw, without the nut. The longest diameter was measured as *a* (mm) with the widest diameter as *b* (mm) (yellow lines). The total area was as S_0_ = a × b (mm^2^). The software Image-Pro Plus was used to calculate the tissue area, which was colored by toluidine blue in this rectangle. The percentage of bone in-growth area was defined as S % =S_1_/S_0_ x 100%.

Rabbits woke up naturally after the surgery, were fed regularly, and raised in the cages (65 cm × 40 cm × 40 cm) to restrict their movement; however, the affected limb was not immobilized. Intramuscular injection of penicillin (4 × 10^5^ U) was performed during the first three days after modeling to prevent infection. Specimens were collected from the two groups of rabbits 3, 6, and 12 weeks after surgery; five rabbits were collected from both groups each time. The bone specimens from the humerus head to the distal end (at least 3 cm) were preserved, and all muscles and soft tissues were removed. Neutral formalin was used for specimen fixation for 48h, followed by dehydration through an alcohol gradient (concentrations: 70%, 80%, 90%) for 7 days, and 100% dehydration twice for 2 days each time. After soaking the specimens in photocurable resin (Technovit7200VLC) for one month, the specimens were performed with a Photocure embedding technique. Then, an EXAKT Hard Tissue Cutting System (EXAKT 400CS/Aw Micro Grinding System) was used to slice the specimen parallel to the shaft of the humerus through the anatomical axis. About 1 mm of bone tissue was reserved on the inside and outside of the axis, respectively. Toluidine blue staining was performed on all of the specimen slices (Figure 4). The image was then reviewed under an optical microscope at 4× magnification. The bone in-growth area was measured and analyzed using Image-Pro Plus software. A rectangle was made on the sagittal section that contained both the long diameter a (mm) and wide diameter b (mm) of the screw (nut removed). The total area was defined as S_0_ (S_0_ = a × b, mm^2^). Thereafter, the bone in-growth tissue area that could be stained with toluidine blue within the rectangle above was calculated using Image-Pro Plus software (S_1_, mm^2^). The percentage of bone in-growth tissue area was defined as S% (S%=S_1_/S_0_×100%) (Figure 5). All histological measurements were performed by the same operator. Figure 4 is a schematic diagram of implant slices with classical regular hexahedron structure.

### Observation index

(1) Qualitative observation: We observed and compared the general situation of plant bone length in different pore structures over time.

(2) Quantitative measurement: We compared the human histological characteristics of bone length of plants in two groups at different time points (3 weeks, 6 weeks, 12 weeks).

### Statistical analysis

SPSS 20.0 software was used for statistical analysis. The data were described as mean ± standard deviation, and the differences of the percentage of bone in-growth areas between the study and the control group were evaluated at the three time points (3 weeks, 6 weeks, 12 weeks). A student t-test was used for analyzing for the continuous variable. An ANOVA analysis was performed to compare the areas at the three time points intra-group, in which *P* < 0.05 was considered statistically significant.
